# Postmortem Diagnosis of Gallbladder Cancer Presenting as Pulmonary Lymphangitic Carcinomatosis With Concurrent Gallbladder Adenomyomatosis

**DOI:** 10.7759/cureus.49988

**Published:** 2023-12-05

**Authors:** Naoko Katsurada, Shuichi Tsukamoto, Masatsugu Yamamoto, Shodai Fujimoto, Atsuhiro Masuda

**Affiliations:** 1 Division of Respiratory Medicine, Department of Internal Medicine, Kobe University Graduate School of Medicine, Kobe, JPN; 2 Division of Pathology, Department of Pathology, Kobe University Graduate School of Medicine, Kobe, JPN; 3 Division of Gastroenterology, Department of Internal Medicine, Kobe University Graduate School of Medicine, Kobe, JPN

**Keywords:** respiratory failure, pulmonary lymphangitic carcinomatosis, autopsy, gallbladder cancer, gallbladder adenomyomatosis

## Abstract

In pulmonary lymphangitic carcinomatosis, it can be difficult to identify the primary site of the cancer on computed tomography (CT) imaging. Here, we report a rare case of pulmonary lymphangitic carcinomatosis, which was difficult to diagnose as gallbladder cancer. An 81-year-old woman, previously followed up for gallbladder adenomyomatosis, presented with persistent cough. CT revealed multiple small nodular opacities, irregular interlobular septal thickening, and bilateral pleural effusions. Based on the CT findings and the presence of malignant cells in the pleural fluid, a presumptive diagnosis of pulmonary lymphangitic carcinomatosis was made, but the primary site was not identified. The patient died of respiratory failure in two months. Autopsy confirmed gallbladder cancer with pulmonary lymphangitic carcinomatosis and multiorgan metastasis. Clinicians should be aware that in patients with gallbladder adenomyomatosis, gallbladder cancer can present with rapidly progressive respiratory symptoms even in the absence of an evident mass or increased gallbladder wall thickening.

## Introduction

Pulmonary lymphangitic carcinomatosis is the infiltration of malignant cells and inflammation of lymphatic vessels due to metastasis of malignancy. The most common underlying primary sites of malignancy coexisting with lymphangitic carcinomatosis are the breast, lung, and stomach [1,2]. Computed tomography (CT) findings show smooth or nodular thickening of interlobular septa and peribronchovascular interstitium and ground-glass opacities [2]. Gallbladder cancer is the most common malignant tumor of the biliary tract and accounts for 0.6% of all newly diagnosed cancers and 0.9% of all cancer-related mortalities [3,4]. Gallbladder adenomyomatosis is a benign disease characterized by excessive epithelial proliferation and muscle hypertrophy of the gallbladder wall. Gallbladder adenomyomatosis is initially evaluated with abdominal ultrasonography, and if the diagnosis is not confirmed by ultrasonography, further evaluation with magnetic resonance imaging (MRI) is recommended.

There is no universal recommendation for the management of asymptomatic gallbladder adenomyomatosis, but follow-up abdominal ultrasonography every three to six months is suggested [5]. Although the clear relationship between gallbladder adenomyomatosis and gallbladder cancer is unknown, gallbladder cancer may occur in cases with gallbladder adenomyomatosis. Differentiating gallbladder cancer from gallbladder adenomyomatosis by using CT or ultrasonography poses diagnostic challenges [6]. Moreover, reports of gallbladder cancer presenting with pulmonary lymphangitic carcinomatosis are limited. There are only two case reports of pulmonary lymphangitic carcinomatosis caused by gallbladder cancer [7,8]. We report a case of postmortem diagnosed gallbladder cancer with pulmonary lymphangitic carcinomatosis in a patient who had been followed up for six years for gallbladder adenomyomatosis.

## Case presentation

**Figure 1:**
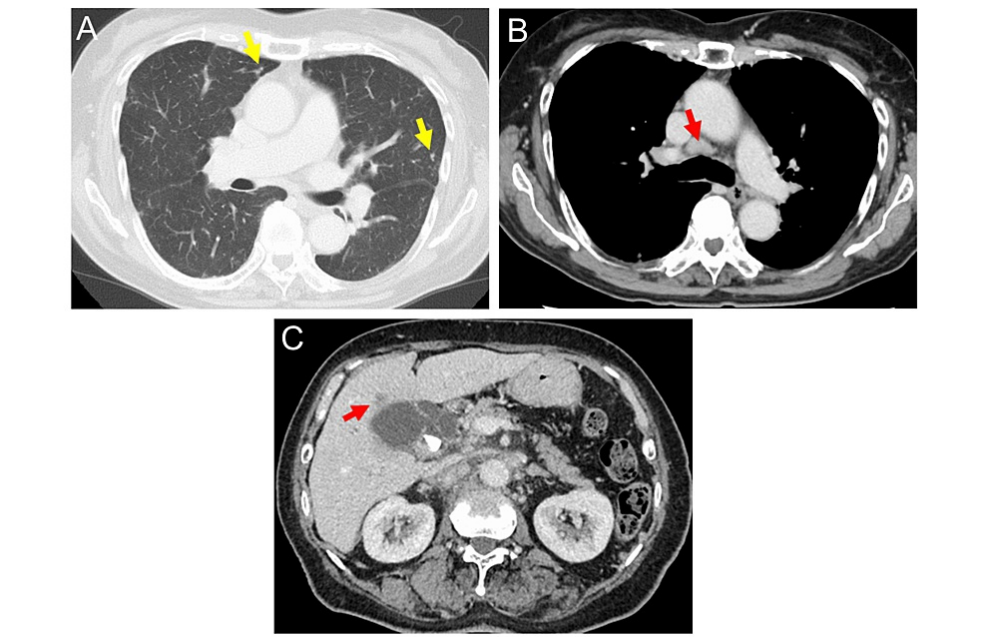
Computed tomography one month prior to admission A) The yellow arrows indicates multiple granular shadows. B) The red arrows indicate enlarged mediastinal and hilar lymph nodes. C) Computed tomography shows thickened gallbladder wall, visible gallstones, and a liver mass. The red arrow indicates the liver mass. A layer is observed between the liver mass and the gallbladder.

An 81-year-old woman with history of gallbladder adenomyomatosis, rheumatoid arthritis, hypertension, hysterectomy for uterine myoma, and bowel obstruction presented to the department of respiratory medicine in our hospital complaining of a persistent cough for two weeks. She had had regular visits with gastroenterology for her gallbladder adenomyomatosis with alternating MRI and abdominal ultrasonography every six months. Over the past six years, these imaging studies consistently showed unchanged gallbladder wall thickening. Contrast-enhanced chest CT showed multiple nodular opacities in the lungs and lymphadenopathy in the cervical, hilar, and mediastinal regions (Figure 1A, 1B). Abdominal CT showed multiple hepatic masses and para-aortic lymphadenopathy (Figure 1C). The gallbladder wall thickening remained unchanged compared to the previous CT.

**Figure 2:**
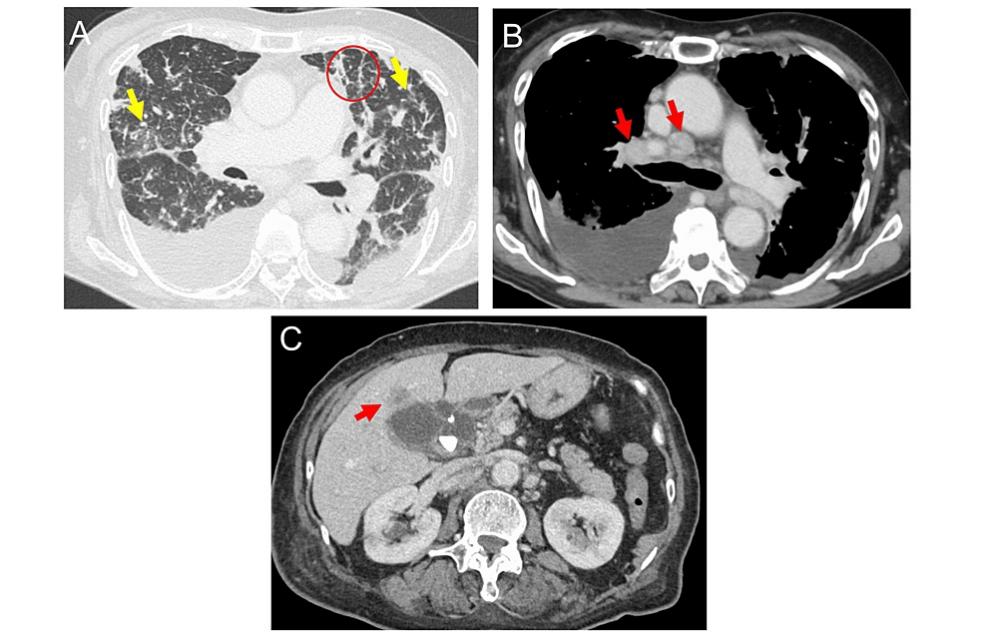
Computed tomography on admission A) The yellow arrows indicate multiple small nodular opacities.  The red circle indicates irregular thickening of the interlobular septa. B) Computed tomography shows further enlargement of mediastinal and hilar lymph nodes (red arrows) and appearance of bilateral pleural effusions. C) Computed tomography shows further enlargement of the liver mass. The red arrow indicates the liver mass. A layer is observed between the liver mass and the gallbladder.

The patient was scheduled for a one-month CT follow-up, as the pulmonary nodules and mediastinal lymph nodes were too small for biopsy. Subsequently, a gastroenterologist assessed the liver mass via abdominal ultrasonography and, finding it suitable for biopsy, decided to proceed with a liver biopsy. The patient was admitted four weeks later for a liver mass biopsy. On admission, percutaneous oxygen saturation was 90% in room air, and fine crackles were audible in the lungs. The Eastern Cooperative Oncology Group performance status was worse, with a score of three. CT findings on admission revealed further enlargement of lymph nodes, and bilateral pleural effusions appeared with multiple small nodular opacities irregular thickening of the interlobular septa and bronchovascular bundles and reticular shadows in both lungs (Figure 2A, 2B). Abdominal CT showed further enlargement of liver mass (Figure 2C).

Laboratory results showed an albumin level of 2.2 g/dL, C-reactive protein level of 10.75 mg/dL (reference range, <0.14 mg/dL), carcinoembryonic antigen level of 2.5 ng/mL (reference range, <5.0 ng/mL), carbohydrate antigen 19-9 level of 7 U/mL (reference range, <37 U/mL), and soluble interleukin-2 receptor level of 1526.3 U/L (reference range, 156.6-474.5 U/mL). Meanwhile, levels of lactate dehydrogenase, aspartate aminotransferase, alanine aminotransferase, alkaline phosphatase, γ-glutamyl transpeptidase, and total bilirubin were all within normal limits. Based on the CT findings of the lungs, we suspected pulmonary lymphangitic carcinomatosis or lymphoproliferative disease. Liver biopsy and bronchoscopy were not performed owing to the deteriorating health condition of the patient. Thoracentesis on the second day of hospital stay revealed adenocarcinoma in the cell block of the pleural effusion, with negative thyroid transcription factor-1 (TTF-1) and p40 staining. Considering the negative immunostaining specific for lung cancer and the absence of pulmonary mass on chest CT, the primary lesion was deemed unlikely to be lung cancer. We considered gallbladder cancer as one of the differential diagnoses and carefully checked the abdominal CT, which showed no mass or further thickening of the gallbladder wall and no evidence of direct invasion to the liver. The patient had no abdominal symptoms.

**Figure 3:**
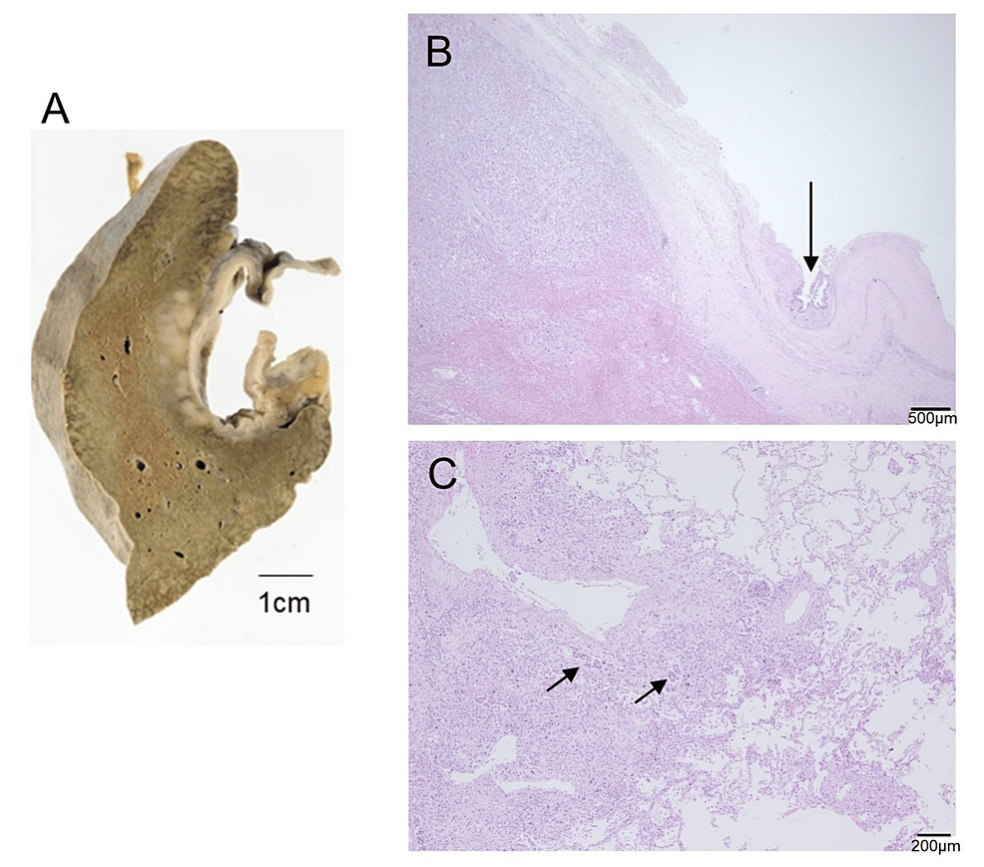
Pathological images of the gallbladder, liver, and lung A) Macroscopic image of the gallbladder and liver. Whitish lesions radiating from the gallbladder to the liver are observed. B) Microscopic image of hematoxylin and eosin staining showing adhesion of the gallbladder and the liver. The gallbladder wall is thickened due to fibrosis and adhered to the liver. The arrow indicates intraepithelial lesion of adenocarcinoma. Note that the liver has adenocarcinoma (upper left). C) Microscopic image of the hematoxylin and eosin staining of the lung. Arrows indicate lymphatic vessel invasion of adenocarcinoma, whose morphology is similar to the lesion in the gallbladder and liver.

A slightly elevated γ-GTP level of 47 was reported on hospital day 20, but other hepatobiliary enzymes were within the normal range during hospitalization. We could not find the origin of cancer. Because of the deteriorating health condition, only palliative treatment was performed, including dexamethasone and morphine hydrochloride, for dyspnea due to presumed pulmonary lymphangitic carcinomatosis. The respiratory condition and chest radiography findings deteriorated rapidly, and the patient died one month after admission due to respiratory failure. After obtaining consent from the patient’s family, pathological autopsy was performed that revealed a poorly differentiated adenocarcinoma with a primary tumor in the epithelium of the gallbladder, which had directly invaded the adjacent liver and bile ducts. Immunostaining of the gallbladder cancer cells was positive for cytokeratin (CK)19 and CK7 and negative for TTF-1. The tumor metastasized to multiple organs, predominantly via the lymphatic system, with lymphangitic carcinomatosis evident in both lungs (Figure 3). The gallbladder was determined to be a primary site of malignancy.

## Discussion

In our case, the primary cancer site remained undetected throughout the patient's life. Post-mortem examination revealed gallbladder cancer as the origin. Although the pleural effusion's cell block indicated adenocarcinoma, extensive CT evaluations during the patient's treatment failed to identify the cancer's primary site. A systematic review reported that 73% of cancers of unknown primary origin can be identified at autopsy, of which the most common were lung (27%), pancreas (24%), and hepatobiliary cancers (8%) [9]. The imaging findings of the gallbladder cancer include mass formation (40-65%), wall thickening (20-30%), and intraluminal polypoid lesions (15-25%) [6]. Differentiating gallbladder cancer from benign conditions in the wall-thickening type is challenging because several benign conditions, including gallbladder adenomyomatosis, also present with the thickening of the gallbladder wall [6]. Moreover, detecting gallbladder cancer concurrent with benign gallbladder diseases remains challenging. The rate of gallbladder cancer complications in biliary adenomyosis is approximately 1-7% [6,10,11]. The stage of gallbladder cancer at diagnosis is more advanced in patients with gallbladder adenomyomatosis than in those without adenomyomatosis [12]. The authors noted that discriminating gallbladder wall thickening from gallbladder cancer in the presence of adenomyomatosis was challenging.

In our case, the patient was regularly followed up for gallbladder adenomyomatosis by abdominal ultrasonography and MRI at the department of gastroenterology; however, premortem detection of gallbladder cancer was difficult because the thickness of the gallbladder wall remained unchanged, with no masses or polyps in the gallbladder. Pathological examination revealed no obvious mass in the gallbladder wall; however, several layers of cancer cells were observed in the gallbladder epithelium. In addition, the absence of abdominal symptoms or an obvious elevation of biliary enzymes further complicated the diagnosis.

To our knowledge, only two cases of pulmonary lymphangitic carcinomatosis due to gallbladder cancer have been reported [7,8]. In one patient who presented with hemoptysis, CT showed diffuse ground-glass opacities and slightly thickened interlobular septa, which were thought to be alveolar hemorrhages; however, post-mortem diagnosis confirmed pulmonary lymphangitic carcinomatosis due to gallbladder cancer. Imaging findings of gallbladder cancer were not present, suggesting difficulties with premortem diagnosis [7]. In another case, there was a mass formation in the gallbladder on CT, and pulmonary lymphangitic carcinomatosis due to gallbladder cancer was considered differential diagnosis; a bronchoscopic biopsy showed cancer cells with the same histological findings as liver metastases, confirming a premortem diagnosis of gallbladder cancer [8]. The tissues in the two cases of gallbladder cancer were poorly differentiated adenocarcinomas, similar to those observed in our case [7,8].

Our patient experienced a relatively rapid disease course. Gallbladder cancer is often considered advanced and unresectable at the time of diagnosis. The reported prognosis for unresectable gallbladder cancer with the best supportive care is 4.5 months [13,14]. Nearly half of patients with pulmonary lymphangitic carcinomatosis die within two months after the onset of respiratory symptoms [1]. Both aforementioned patients with pulmonary lymphangitic carcinomatosis due to gallbladder cancer died within 30 days of admission [7, 8]. Consistent with these reports, our patient died approximately 2.5 months after symptom onset.

## Conclusions

This is a rare case of pulmonary lymphangitic carcinomatosis due to gallbladder cancer. Clinicians should recognize that patients with gallbladder cancer may present with pulmonary lymphangitic carcinomatosis that deteriorates rapidly. Furthermore, the absence of a mass or wall thickening in gallbladder adenomyomatosis does not exclude the presence of gallbladder cancer.
